# Mechanism of injury and management in traumatic anterior shoulder dislocation with concomitant humeral shaft and ipsilateral scapula fracture: a case report and review of the literature

**DOI:** 10.1186/1752-1947-8-431

**Published:** 2014-12-16

**Authors:** Kamran Farooque, Kavin Khatri, Chaitanya Dev, Vijay Sharma, Babita Gupta

**Affiliations:** 18 Department of Orthopaedics, Jai Prakash Narayan Apex Trauma Centre, All India Institute of Medical Sciences, AIIMS, Ring Road, New Delhi, Delhi 110 029 India; 19 Department of Anaesthesia and Critical Care Medicine, Jai Prakash Narayan Apex Trauma Centre, All India Institute of Medical Sciences, AIIMS, Ring Road, New Delhi, Delhi 110 029 India

**Keywords:** Shoulder dislocation, Scapular fracture, Humeral fracture

## Abstract

**Introduction:**

Traumatic anterior dislocation of the shoulder is an emergency and warrants urgent attention. However, it becomes difficult to manage in cases of associated fractures of humerus and other bones surrounding the shoulder joint. There have been reports of traumatic anterior dislocation of the shoulder associated with humeral fractures in the literature but the trilogy of anterior dislocation of the shoulder; humeral shaft fracture; and scapular fracture have never been described.

**Case presentation:**

We present the case of a 27-year-old south Asian man presenting with the above-mentioned injury. He was managed with open reduction and internal fixation of the fracture and subsequent reduction at the shoulder joint. The fracture of the scapula was managed conservatively. Radiological union was achieved at 14 weeks with a good range of movements at the shoulder.

**Conclusions:**

Shoulder dislocation associated with fractures of humerus and scapula occurs in rare circumstances due the peculiar mechanism of injury. There is risk of neurovascular damage while attempting joint reduction without fracture fixation so, in these cases, the fracture should be addressed first and dislocation later.

## Introduction

Traumatic anterior dislocation of the shoulder with concomitant humeral shaft and ipsilateral scapula fracture is a serious but rare injury. There have been reports of anterior dislocation with humeral fracture in the literature, but its association with fracture of the scapula has not been reported. We report a case of traumatic anterior dislocation of the shoulder, associated with ipsilateral fracture of shaft humerus along with fracture of the scapula due to an unusual mechanism of injury and its proposed management.

## Case presentation

A 27-year-old, right-hand-dominant south Asian man sustained a road traffic accident and presented to our emergency department four hours after the accident. He was walking along the road, when he was hit by a motor vehicle (a truck) from behind. He fell by the side of the road and landed with his left limb trapped under his body.

**Figure 1:**
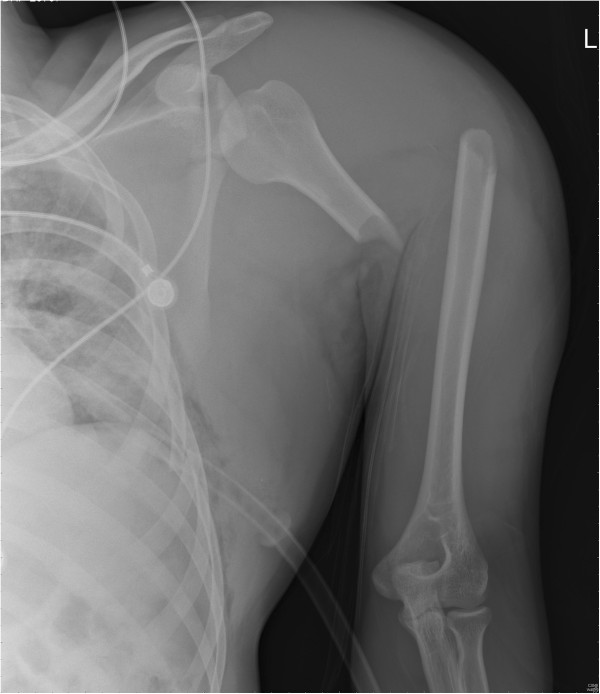
**Radiograph showing fracture of humeral shaft, shoulder dislocation and scapular fracture.**

**Figure 2:**
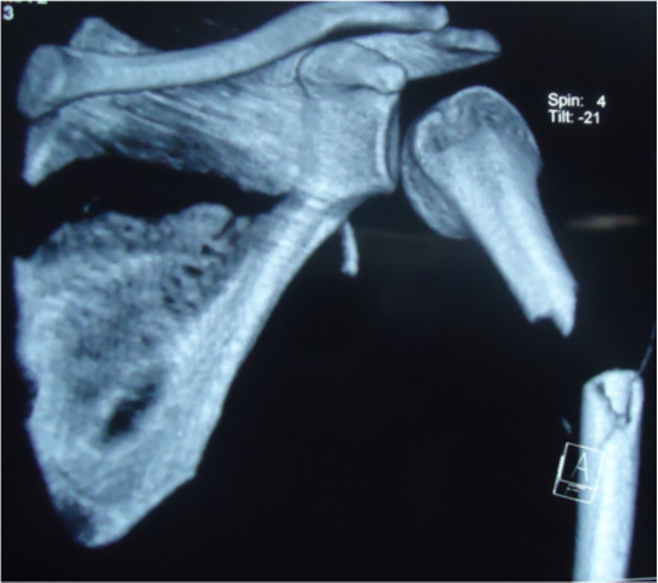
**Computed tomography (virtual reconstruction) outlining the scapular fracture pattern.**

**Figure 3:**
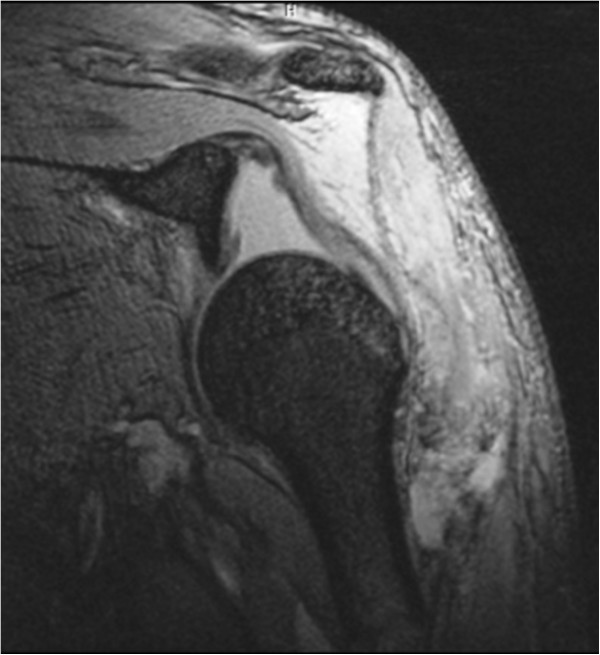
**Coronal section of magnetic resonance imaging showing shoulder dislocation and intact rotoator cuff.**

**Figure 4:**
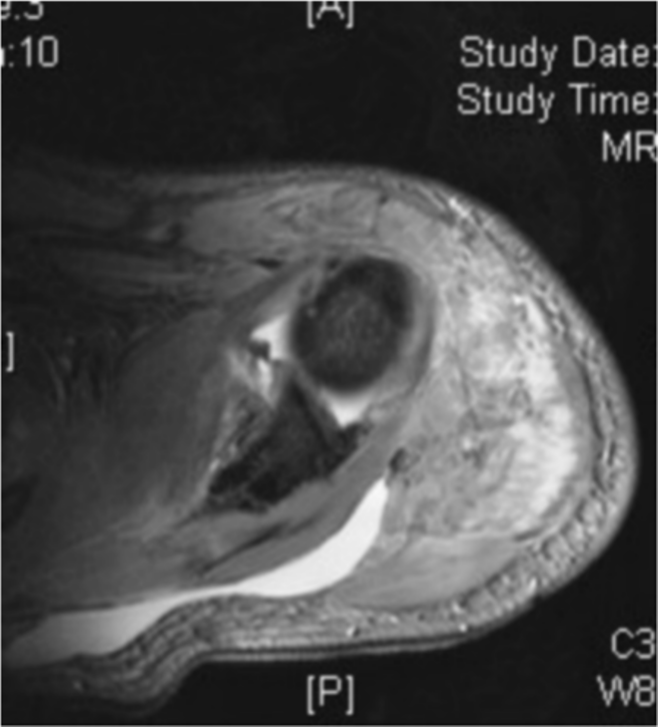
**Saggital section of magnetic resonance imaging revealing labral and bicipital tear.**

On arrival, he was conscious and well oriented with stable vital signs but complained of breathing difficulty. He also complained of pain in his left shoulder and arm. On examination, the air entry was found to be decreased on the left side of his chest. Radiography of his chest was done and a diagnosis of hemopneumothorax was made by the emergency physician. A chest tube was inserted in his left hemithorax and 150ml of blood was drained. There was relief in his breathing difficulty and air entry was found to be equal on both sides of his chest. There was visible deformity of the left humerus, with abrasions over the left scapular region, and no distal neurovascular deficit in the left upper limb. Local examination revealed a positive sulcus sign and anteriorly displaced humeral head along with unnatural mobility at the humeral shaft. A radiographic examination confirmed an anterior dislocation of the shoulder, with transverse fracture of the shaft humerus at the proximal one-third and distal two-third junction, along with fracture of the ipsilateral scapula and left-sided third, fourth and fifth rib fractures (Figure 1). A computed tomography scan was done to outline the scapular fracture pattern, and decide its management (Figure 2). A magnetic resonance imaging scan of the shoulder revealed a tear in the anteroinferior and superior labrum, a partial bicipital tear and no injury to the rotator cuff (Figures 3 and 4).

**Figure 5:**
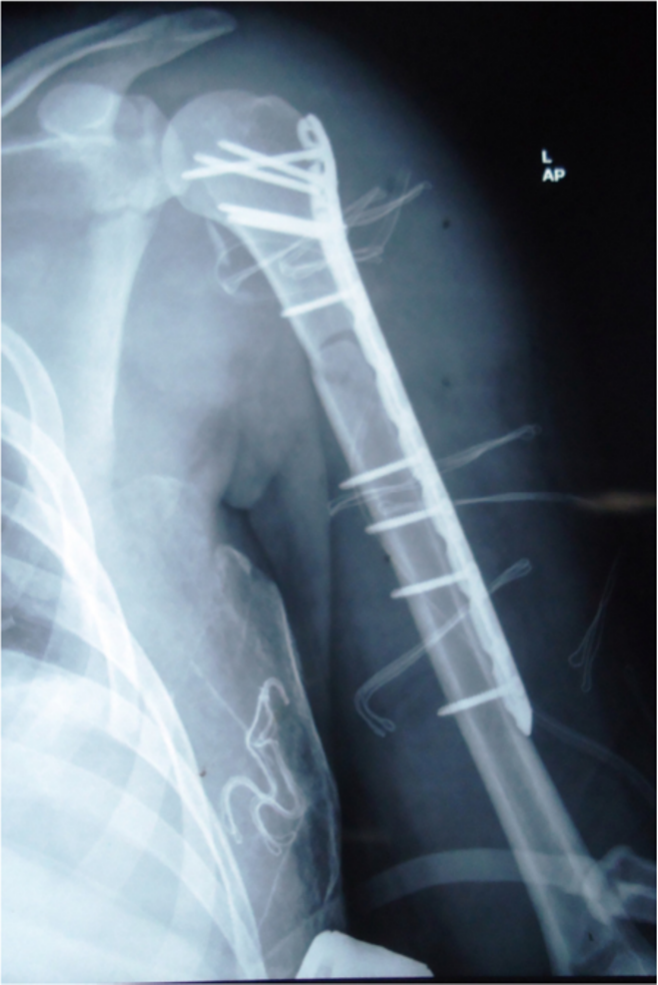
**Reduced shoulder and acceptable fracture fixation with proximal humerus locking plate.**

A closed reduction of the shoulder dislocation was not attempted due to the concomitant humeral shaft fracture, with high risk of failure and iatrogenic neurovascular damage [1]. The fracture fixation should be carried out prior to joint reduction to avoid neurovascular traction injury. Following a secondary survey, our patient was transferred to the operating room and general anesthesia was administered. The humerus was approached through an extended deltopectoral approach. The humeral shaft fracture was reduced and fixed with a proximal humerus locking plate. The shoulder was subsequently reduced by gentle manipulation, under direct vision. The rotator cuff was examined for its integrity and was found to be intact, which reinforced the magnetic resonance findings. The labral repair was deferred due to the prolonged anesthesia time. The scapular fracture was managed conservatively as the fracture pattern did not warrant a surgical intervention. Postoperative radiographs confirmed a concentric reduction of the shoulder joint and good fracture fixation (Figure 5). Our patient did not have any neurovascular deficit post surgery.

**Figure 6:**
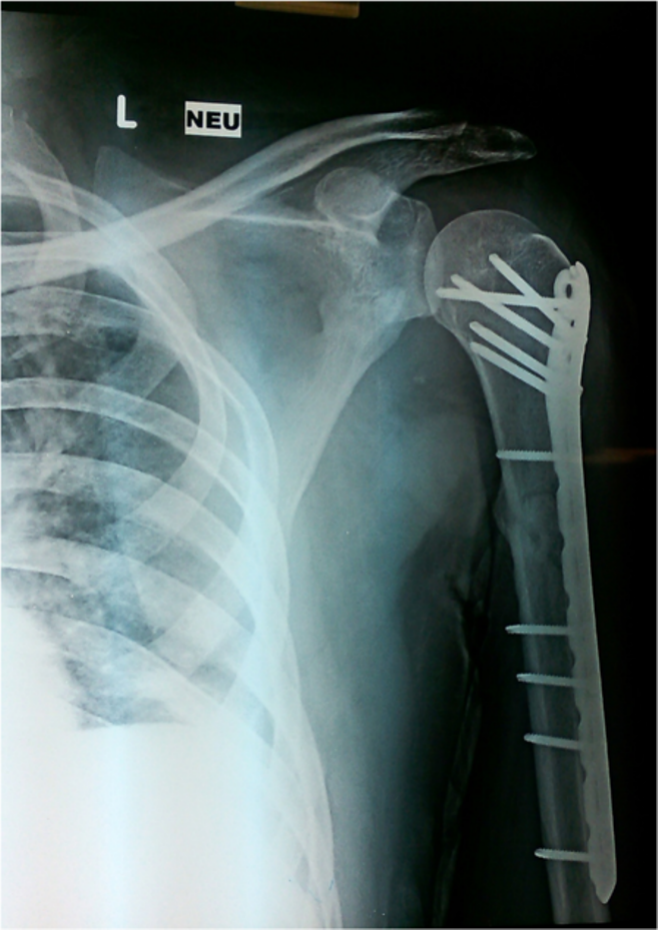
**Radiograph showing callus formation at the fracture site.**

**Figure 7:**
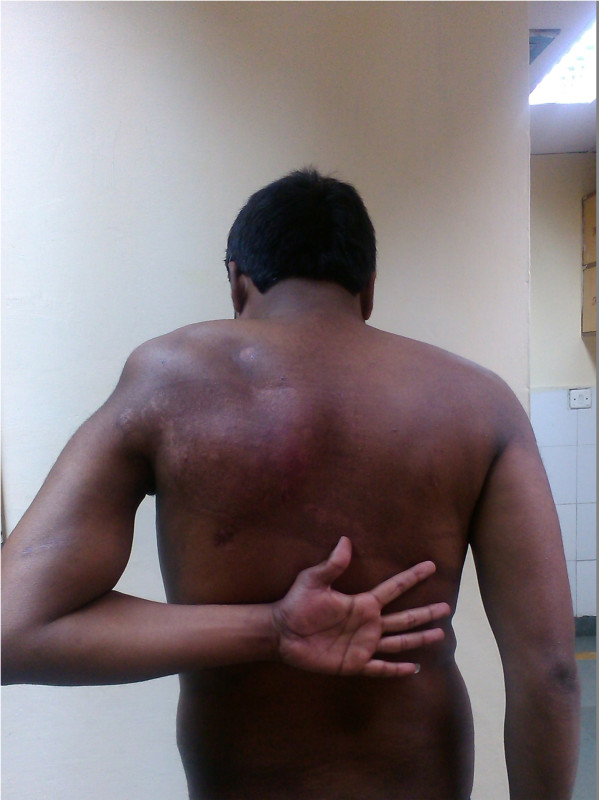
**Internal rotation at the shoulder joint at six months after operative intervention.**

**Figure 8:**
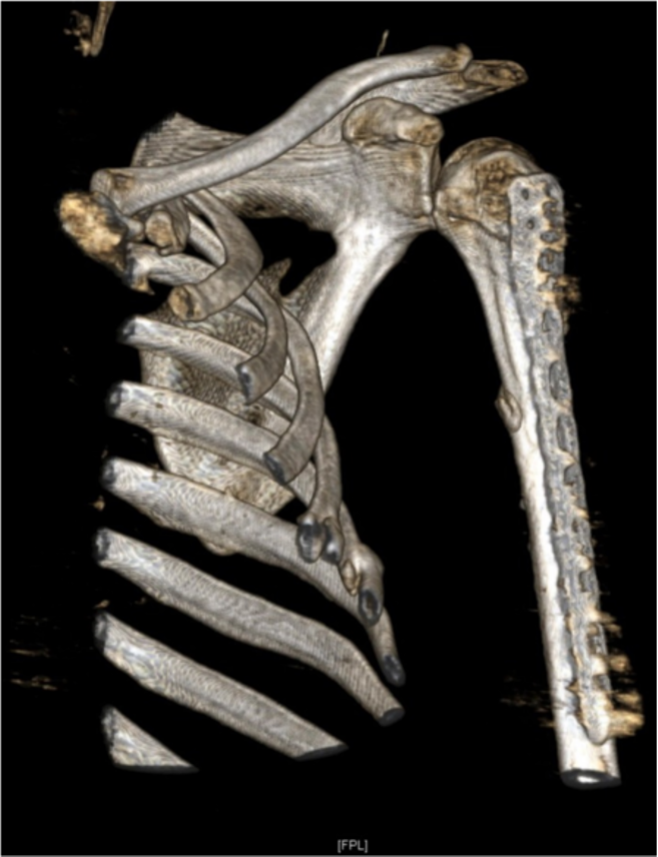
**Computed tomography scan showing radiological union of scapular and humeral fracture.**

Our patient was given an arm sling and pendulum exercises for the involved shoulder along with a range of motion exercises at the elbow joint that were initiated in the immediate postoperative period. The recovery of our patient was uneventful and the chest drain was removed on the third postoperative day. Our patient was discharged on the fourth postoperative day. After three weeks, active assisted flexion and external rotation to neutral were allowed. At six weeks, further movements at the shoulder were encouraged. Radiological union at the fracture site was noticed at 16 weeks (Figure 6) with a good range of movements at the shoulder at six months (Figure 7). A follow-up computed tomography scan (Figure 8) of the shoulder revealed union at the fracture site with the humeral head well placed into the glenoid cavity and union at the medial border of the scapula.

## Discussion

Dislocation of the shoulder with ipsilateral humeral shaft fracture is a rare injury with very few reported cases in the literature. The very first case of the same was reported by Winderman *et al.* [2].

Various mechanisms of injury have been proposed by previous authors. Whether the dislocation or the fracture occurs first is debatable. Some authors have postulated that direct transmission of force leads to simultaneous dislocation and fracture [3] while others propose, indirect force leading to the dislocation and direct force leads to the fracture. Kontakis *et al.* [4] proposed dislocation at the shoulder was the preceding event and subsequently the bending or torsional force fractured the shaft of the humerus while Sankaran-Kutty *et al.* [5] postulated that force along the axis of the humerus resulted in dislocation and fracture of the humeral shaft. In our case, our patient was hit from behind by a speeding truck while he was a pedestrian. The force was transmitted from back to front, which resulted in the fracture of his scapula and the anterior dislocation of his shoulder. The same force vector may have fractured the humeral shaft or the shaft could have been fractured secondary to the fall with the left arm entrapped under the body, as a result of direct impact with the ground.

The treatment modalities include closed reduction and plaster of paris slab application [6], nailing [7], and plating [8] of the humeral shaft along with reduction of the shoulder dislocation. Good clinical results have been reported with almost all the modalities. However, there is no consensus in the literature for treating this type of injury.

In our case, the fracture of the humeral shaft was treated by open reduction and internally fixed with a proximal humerus locking plate as the transverse fracture was located in the proximal fourth of the humeral shaft. A dynamic compression plate would not have been able to engage a minimum eight cortices in the proximal fragment as per AO recommendation. Subsequently, the anterior dislocation of the shoulder was reduced, with gentle manipulation intraoperatively, under direct vision. Zlowodzki *et al.* [9] in their systematic review of scapular fractures had reported excellent/good results in 82% of the cases treated conservatively. Cole *et al.* [10] had suggested operative intervention in scapular fractures with a glenopolar angle greater than 30 degrees, medial displacement of the lateral border by more than 25mm, angular deformity more than 45 degrees, and concomitant intraarticular step greater than 3mm or double-displaced disruption of the superior shoulder suspensory complex. In our case, the scapular body fracture did not meet any of the criteria suggested by Cole *et al.* [10] for operative intervention. Therefore the scapular body fracture was managed conservatively. The fracture united with good functional outcome at both the glenohumeral and scapulothoracic joint.

## Conclusions

Dislocation of the shoulder is rarely associated with diaphyseal fracture of the humerus and fracture of the ipsilateral scapula. The anteriorly direct force and subsequent fall creating a second force vector could have led to the fracture dislocation. Prompt treatment with open reduction and internal fixation of the humeral fracture helps in reducing the shoulder dislocation thereby improving the functional outcome at the shoulder joint. The joint reduction should not be attempted prior to fracture fixation due the high risk of neurovascular damage. The scapular fracture needs to be treated on its own merit, depending on the fracture configuration.

## Consent

Written informed consent was obtained from the patient for publication of this case report and any accompanying images. A copy of the written consent is available for review by the Editor-in-Chief of this journal.
